# Metal 3D printing as a disruptive technology for superalloys

**DOI:** 10.1038/s41467-020-16188-7

**Published:** 2020-05-11

**Authors:** Chinnapat Panwisawas, Yuanbo T. Tang, Roger C. Reed

**Affiliations:** 1 Department of Materials, University of Oxford, Parks Road, Oxford, OX1 3PH UK; 2 NISCO UK Research Centre, School of Engineering, University of Leicester, Leicester, LE1 7RH UK

**Keywords:** Mechanical properties, Metals and alloys

## Abstract

3D printing can allow for the efficient manufacturing of elaborate structures difficult to realise conventionally without waste, such as the hollow geometries of nickel-based superalloy aeronautic components. To fully exploit this method, we must move towards new alloys and processes.

## Conventional superalloy manufacturing

Superalloys, a family of metal mixes based on nickel, cobalt, or iron, are resistant to high temperature deformation, corrosion and oxidation, particularly when operating at elevated temperature close to their melting point. They were first developed for gas turbine components in turbojet engines, and are now widely used for high temperature applications in the aerospace and power generation industries. To achieve these high temperature properties (both mechanical and chemical), microstructural control is critical and is enabled by a combination of specific alloying element additions and careful manufacturing processes.

Nickel-based superalloys, the earliest and best-developed superalloy family, rely on a two-phase microstructure consisting of a strengthening phase—a dispersion of (Ni,Co)_3_(Al,Ti,Ta) precipitates (of L1_2_ crystallography) called γ′—grown in a matrix of Cr-enriched Ni. Other alloying elements such as refractories (Re, Mo, W) or metalloids (B, C) may also be added. Based on their chemistry, these alloys are some of the most complex humanity has designed. During conventional processing, this crucial precipitation occurs via a diffusion-controlled reaction during cooling in the temperature range 1000–750 °C^1^.

Manufacturing is traditionally the ‘Achilles’ heel’ of superalloy applications—structurally sound mechanical properties have not been achieved without long-winded and costly subtractive manufacturing via machining of castings. Today, we still use precision investment casting processes that date back to classical antiquity. For example, to produce a jet engine turbine blade, both a wax model and silica-based replica of the cooling channels are needed to create a ceramic mould for every component produced, into which kilograms of molten metal are cast under vacuum. Cooling to ambient conditions takes several hours, and it is impossible to suppress the precipitation of the γ′ precipitates during cooling; moreover, very careful subsequent heat treatment of several hours at ~1300 °C is needed—just below the melting temperature—to reduce chemical dendritic segregation from the casting route. Finally, machining is required to shape the final intricate turbine blade geometry. The investment casting process involves several chemical and process controls with significant waste/scrappage generated during the casting and subsequent machining of the turbine parts: only about 10% of the superalloy ends up as finished goods^2^.

## 3D printing as a new processing avenue for superalloys

Using 3D printing, or additive manufacturing (AM), instead of investment casting allows processing to occur radically differently, with reduced manufacturing steps and minimum processing waste. The laser-based melting and consolidation of solid powder of a few tens of microns in diameter, layer-by-layer, under direct input from a computer-aided design (CAD) system, confers an as-of-yet untapped freedom of design: hollow structures, foam-like or lattice-based architectures, with more effective use of materials in an additive as opposed to subtractive way. In addition, the AM process, with its melting and re-melting of fine powder size in micron length and time scale, leads to high cooling rates of 10^3^–10^6^ °C/s and a very different metallurgical response to processing^3^. Solidification gives rise to a very fine cellular rather than dendritic microstructure^4^, which virtually eliminates the dendritic segregation found in conventional processing, removing the need for a chemical homogenisation step. The precipitation of γ′ is also suppressed by the severe cooling rate, allowing for nano-scale precipitation to be tailored during subsequent heat treatment for improved properties^5^. The precipitation phase can be optimised by designing new heat treatment protocols to obtain desirable microstructures associated with high strength in AM superalloys^6^.

**Fig. 1:**
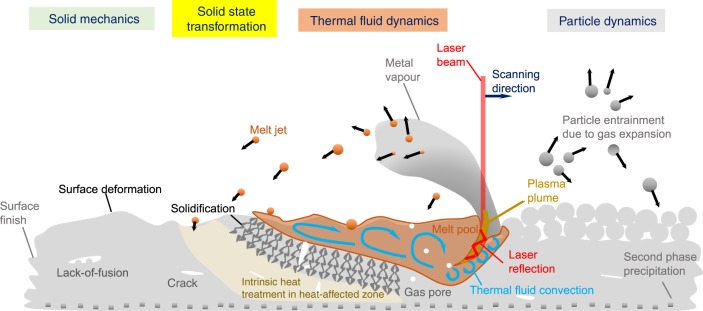
A schematic illustration of multi-scale, multi-physics phenomena in powder-bed fusion AM. Different physical effects and associated physics taken place during AM include powder particle dynamics due to gas expansion, thermal fluid dynamics capturing solid-liquid-vapour transition when interacted with laser, solid state transformation such as precipitation once remelting and intrinsic heat treatment, and subsequent solid mechanics to deal with damage mechanism such as cracking.

However, widespread application of AM in superalloys for complex hollow structures such as aero-jet turbine blades is still not straightforward. In order to successfully leverage AM techniques in superalloys, we need an improved understanding of the science of the process; many aspects of it are obscure because the fundamentals of AM involve multiple physical and chemical phenomena across length and time scales (see Fig. 1). For instance, when the laser comes into contact with the metal powder, all possible four states of matter—solid, liquid, gas vapour and plasma—interact^7^, and very few if any physics-based models exist to address this complexity. In addition, the nature of the rapid and repeated thermal cycles induces intense thermal gradients and thus chemical, structural and mechanical states which are metastable, triggering metallurgical defects^8^ which jeopardise properties^9^.

Finally, most conventional superalloys cannot be readily migrated from investment casting to 3D printing because they have been optimised for specific processing routes, e.g. forging, welding and casting. Due to the rapid and repeated thermal cycling of the AM process, new compositions that take advantage of these processing parameters can be designed via a computational composition-process data-driven approach to tailor microstructure and properties for AM cooling rates^3^. Novel grades of superalloys optimised for 3D printing and designed to mitigate metallurgical defects such as porosity and cracking^10^ in critical high-temperature components are therefore key to successful commercial take-up.

## Materials and manufacturing design for metal 3D Printing

We envisage carefully prescribed computer-aided design models along with model-based designer alloys and optimised—and indeed spatially varying—3D printing strategies to achieve high-value added components^11^. One can imagine that these will be printed locally, provided that the 3D printing infrastructure needed is distributed (rather than centralised) and input materials are available.

**Fig. 2:**
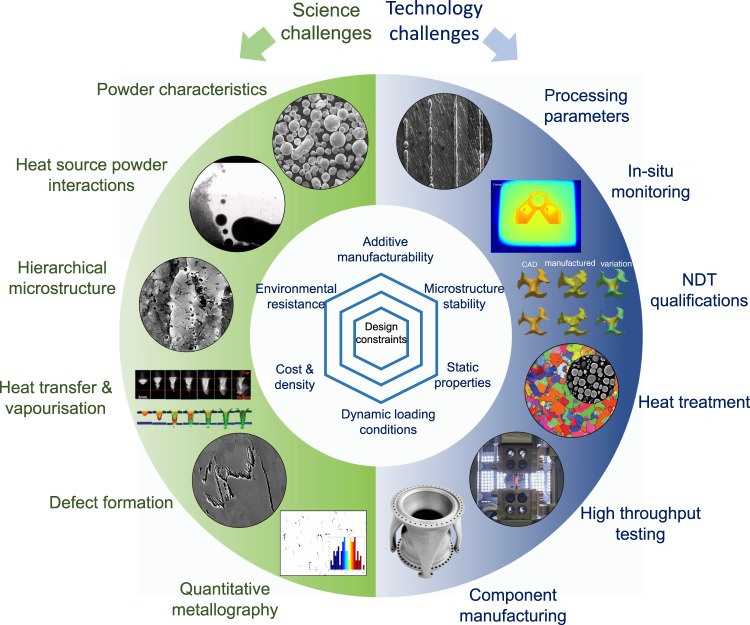
Key challenges in science and technology for digitally designed metal 3D printing, after^7,13–15^. Fundamental understanding of the processing science includes powder flowability and powder shape distribution, interaction with heat source, hierarchical microstructure formed, defects mitigation and better quantification of metallurgical features. Challenges on the technology perspective includes parametric optimisation of processing, real-time monitoring, establishment of qualification standard, high throughput testing and manufacturing of scaled-up components. The design of superalloys for AM has to balance between manufacturability, mechanical integrity, stability and cost.

The challenges are both scientific and technological; some critical ones are highlighted in Fig. 2. These are best addressed using data-driven approaches to account for the complex processing parameters from metal powder characteristics to large degree of freedom of printing strategy which respect the advances being made in data science, physics-based modelling, process modelling, and artificial intelligence^12^. The more technical aspects of AM such as the starting powder and the processing strategy are crucial for component consistency in terms of defect mitigation^13^ and quality assurance. While less fundamental, commercially implementing the AM process will necessitate multi-scale process modelling, improved in-situ monitoring^14^ and post-fabrication treatments, and the adoption of comprehensive industrial standards, especially because these alloys are designed for use in mission-critical applications in the aeronautics and space sectors.

With all these in mind, the materials design approach for superalloy AM requires the use of data from powder processing to melt and printing strategies to post-heat treatments—all in the name of consciously designing both the composition and processing route to achieve minimal defects, minimum waste, and desirable microstructure-properties relationships. Such an approach to manufacturing would allow for a more thoughtful and efficient method to engineer high-performance structural metallic components while respecting the needs of the environment and promoting sustainability.
